# Simiao Qingwen Baidu decoction inhibits Epstein–Barr virus-induced B lymphoproliferative disease and lytic viral replication

**DOI:** 10.1080/13880209.2021.1934038

**Published:** 2021-06-22

**Authors:** Xianhui Yang, Lingling Liu, Huijuan Zhang, Xiaoxu Sun, Yongbin Yan, Ruiying Ran

**Affiliations:** a Graduate School, Henan University of Traditional Chinese Medicine, Zhengzhou, PR China; b Pediatric Zone 5, First Affiliated Hospital of Henan University of Traditional Chinese Medicine, Zhengzhou, PR China

**Keywords:** Proliferation, apoptosis, CGM1 cells

## Abstract

**Context:**

Simiao Qingwen Baidu decoction (SQBD), a traditional Chinese medicine prescription, can ameliorate Epstein–Barr virus (EBV) induced disease. However, its mechanism still remains unknown.

**Objective:**

To detect the mechanism of SQBD in EBV-induced B lymphoproliferative disease *in vitro*.

**Materials and methods:**

Sprague–Dawley (SD) rats (*n* = 20) were given SQBD (10 mL/kg) by gavage once a day for 7 d. SQBD-containing serum was obtained from abdominal aortic blood of rats, and diluted with medium to obtain 5%, 10% or 20%-medicated serum. SD rats (*n* = 10) were given normal saline, and normal serum was collected as a control. EBV-transformed B cells (CGM1) were cultured in medium containing 5%, 10% or 20%-medicated serum. CGM1 cells were treated with normal serum as a control. Cell viability and apoptosis were examined. The expression and activity of proteins were assessed.

**Results:**

We found that IC_50_ (83 ± 26.07%, 24 h; 69.88 ± 4.69%, 48 h) of 10% medicated serum was higher than that of 5% (25.47 ± 6.98%, 24 h; 21.62 ± 7.30%, 48 h) and 20%-medicated serum (51 ± 7.25%, 24 h; 56.03 ± 2.56%, 48 h). Moreover, SQBD promoted apoptosis of CGM1 cells by regulating EBV latency proteins expression. SQBD inhibited EBV-induced lytic viral replication.

**Conclusions:**

Our data confirmed that SQBD inhibits EBV-induced B lymphoproliferative disease and lytic viral replication. This work provides a theoretical basis for the mechanism of SQBD in EBV-induced B lymphoproliferative disease, and SQBD may be an effectively therapeutic drug for EBV-induced B lymphoproliferative disease.

## Introduction

Epstein–Barr virus (EBV) is a lymphotropic virus of the herpesviruses family. EBV infects humans widely and continuously, and it is the pathogen of infectious mononucleosis (Zhang, Li, et al. 2018; Zhang, Nie, et al. 2018). EBV infection leads to immortalized B lymphoblast cell lines. EBV infection is closely associated with the occurrence of nasopharyngeal carcinoma, gastric carcinoma and Burkitt’s lymphoma (Wang et al. 2012; Li et al. 2019; Yoon et al. 2019). The genome of EBV has been detected in lung cancer and breast cancer tissues (Hong et al. 2019; Golrokh, Mofrad et al. 2020). Target cells are infected by EBV in the form of latent, lytic or defect infection. During the state of latent infection, the virus DNA integrates into the human genome or exists as a cyclic molecule in a free state. It often occurs in healthy adults, with no uncomfortable symptoms. Under certain conditions, the infectious viral particles are released after proliferation, activation, immediate-early and early proteins expression, DNA and structural protein replication and assembly. Previous study shows that EBV is closely associated with the occurrence of lymphomas in the patients with immunodeficiency (Sun and Cesarman 2011).

In the early stages of infection, EBV invades lymphoid tissues through oral mucosa. The immortalized B-cells of rest state differentiate into lymphoblastoid and exist in an inactive state in the cells (Martin et al. 1994). The main EBV gene products include EBV nuclear antigen (EBNA), latent membrane protein (LMP) and EBV with small RNA (EBER). Most of them are closely associated with the EBV replication, and few gene products are related to the transformation and immortalization of EBV (Geng and Wang 2015; Grywalska and Rolinski 2015). During the latent state, all the gene products have a carcinogenic effect. LMP1, LMP2A, LMP2B and EBNA1 induce malignant transformation of cells and lead to the occurrence of diffuse large B-cell lymphoma (Brown et al. 2001).

Simiao Qingwen Baidu decoction (SQBD) is a Chinese traditional medicine that has as the major components: gypsum fibrosum, *Anemarrhena asphodeloides* Bunge (Agavoideae), cornu bubali, *Acutellaria baicalensis* Georgi (Lamiaceae), *Rehmannia glutinosa* (Gaertn.) DC. (Orobanchaceae), *Paeonia obovata* Maxim. (Paeoniaceae), *Scrophularia ningpoensis* Hemsl (Scrophulariaceae), *Forsythia suspense* (Thunb.) Vahl (Oleaceae), *Paeonia suffruticosa* Andr., *Platycodon grandiflorus* (Jacq.) A. DC. (Campanulaceae), *Lonicera japonica* Thunb. (Caprifoliaceae), *Angelica sinensis* (Oliv.) Diels (Apiaceae), *Prunella vulgaris* Linn. (Lamiaceae), *Bupleurum chinense* DC. (Apiaceae) and *Glycyrrhiza uralensis* Fisch. ex DC (Fabaceae). SQBD is reported to have an anti-inflammatory effect on various acute and chronic inflammatory diseases (Yu et al. 2014). Previous research shows that SQBD has the pharmacological functions of anti-inflammatory, anticoagulant, protecting blood vessels, improving the blood circulation, inhibiting thrombosis and promoting fibrinolysis (Yan et al. 2016). However, the effect of SQBD on B lymphoproliferative disease and lytic viral replication is still unknown. Thus, we aimed to determine the mechanism of action of SQBD in EBV-induced B lymphoproliferative disease and lytic viral replication *in vitro*.

## Materials and methods

### Experimental animals

Sprague–Dawley (SD) male rats (*n* = 30), 6-weeks-old, were purchased from the Laboratory Animal Centre of Henan University of Traditional Chinese Medicine. All mice were housed in a standard laboratory environment (21 ± 1 °C; 45–55% humidity; 12 h light/dark cycle; free access to food and water). These rats were randomly divided into two groups: experimental group (20 rats) and control group (10 rats). The rats in experimental group were given SQBD (300 mg/mL and 10 mL/kg) by gavage once a day for 7 d. The rats in control group were given the same dose of normal saline. We obtained the blood from the abdominal aorta of rats at 2 h after gavage on the 7th day. The drug-medicated serum and blank serum were obtained from the blood after separation and sterilization.

SQBD contained: gypsum fibrosum (30 g), *Anemarrhena asphodeloides* (10 g), cornu bubali (15 g), *Acutellaria baicalensis* (10 g), *Rehmannia glutinosa* (10 g), *Paeonia obovata* (10 g), *Scrophularia ningpoensis* (10 g), *Forsythia suspense* (10 g), *Paeonia suffruticosa* (10 g), *Platycodon grandiflorus* (6 g), *Lonicera japonica* (10 g), *Angelica sinensis* (10 g), *Prunella vulgaris* (10 g), *Bupleurum chinense* (12 g) and *Glycyrrhiza uralensis* (6 g). SQBD was obtained from the First Affiliated Hospital of Henan University of Traditional Chinese Medicine. In brief, the medicine materials were soaked in water, decocted twice, concentrated, filtered and sterilized to obtain SQBD at a concentration of 300 mg/mL.

All protocols were authorized by the Ethics Committee of First Affiliated Hospital of Henan University of Traditional Chinese Medicine.

### Cell culture and drug treatment

EBV-transformed B cells (CGM1) were obtained from commercial cell banks (ATCC, Manassas, VA). CGM1 cells were cultured in Dulbecco’s modified eagle medium (DMEM) (Sangon Biotech, Shanghai, China) containing 1% penicillin/streptomycin. CGM1 cells were incubated with 5%, 10% or 20% the drug-medicated serum or blank serum. 10% foetal bovine serum (FBS) treated CGM1 cells served as negative control (NC). The CGM1 cells were incubated in a humidified atmosphere at 37 °C and 5% CO_2_.

### CCK8 assay

CCK8 assay was performed to explore the cell viability of CGM1 cells at 24 and 48 h after drug treatment. The cells were suspended in DMEM containing 10% FBS and the cell density was adjusted to 1 × 10^5^/mL. The cell suspension was seeded into 96-well plates, with 100 μL cell suspension in each well. CCK8 reagent (10 μL) was added into each well, and the cells were cultured for 4 h in an incubator at 37 °C. The absorbance of samples was detected at 450 nm using enzyme-labelled instrument (Thermo Fisher Scientific, Waltham, MA).

### Flow cytometry

The CGM1 cells were collected by centrifuging for 5 min at 500 *g*, 4 °C. The cells were washed with pre-cooling PBS for two times. Cells were then resuspended in the Annexin V binding buffer. The cell suspension was dyed with Annexin V-FITC and PI and placed darkness at room temperature for 15 min. Then the cell suspension was mixed with Annexin V Binding buffer and put on ice. The apoptosis rate of cells was determined by flow cytometry in 1 h. The assay was performed according to the instruction of Annexin V-FITC/PI Cell Apoptosis Detection Kit (TransGen Biotech, Beijing, China).

### Western blot (WB)

Total protein was extracted from cells using Tissue or Cell Total Protein Extraction Kit (Sangon Biotech). Equivalent protein from different samples was separated by 10% SDS-PAGE protein electrophoresis, following by transformation onto PVDF membranes (Merck Millipore, Billerica, MA). The membranes were blocked with 5% skim milk at room temperature. After that, the membranes were incubated with primary antibodies, p53 (1:1000, Proteintech, Wuhan, China), EBNA2 (1:500, Abcam, Cambridge, MA), LMP1 (1:500, Abcam), EBNA3A (1:2000, Abcam), EBNA3C (1:1000, Eterlifer, Birmingham, UK), p65 (1:1000, Proteintech), p-p65 (1:1000, Abcam), p52/p100 (1:500, Proteintech), c-MYC (1:1000, Proteintech), cyclin E (1:250, Santa Cruz Biotechnology, Santa Cruz, CA), BZLF1 (1:500, Santa Cruz Biotechnology), BMRF1 (1:3000, Merck Millipore) or p18 (1:2000, Thermo Fisher Scientific) at 4 °C overnight. After the membranes were washed with TBST for several times, secondary antibodies labelled with horseradish peroxidase were incubated with the membranes. β-actin antibody (1:2000, Proteintech) was used as a reference protein for normalization. The grey level of the protein bands was examined by Image J software (Bethesda, MD).

### Detection of caspase 3/7 activity

The activity of caspase 3/7 was detected using Caspase-Glo^®^ 3/7 Assay (Promega Corporation, Madison, WI). The cell supernatant was mixed with equal volume of Caspase-Glo^®^ Reagent. The 200 μL mixture was added into the 96-well plate and incubated at room temperature for 1 h. The fluorescence intensity was detected by GloMax^®^ Navigator System (Promega Corporation, Madison, WI). The assay was performed according to the manufacturers’ instruction.

### Statistical analysis

All experiments were independently repeated at least three times. All values were exhibited as mean ± standard deviation and analysed by SPSS version 22.0 statistical software (IBM, Armonk, NY). For comparison of two groups, a two-tailed Student’s *t*-test was used. Comparison of multiple groups was made using a one- or two-way ANOVA. Difference was considered statistically significant at *p* < 0.05.

## Results

### SQBD promotes apoptosis of CGM1 cells

**Figure 1.**
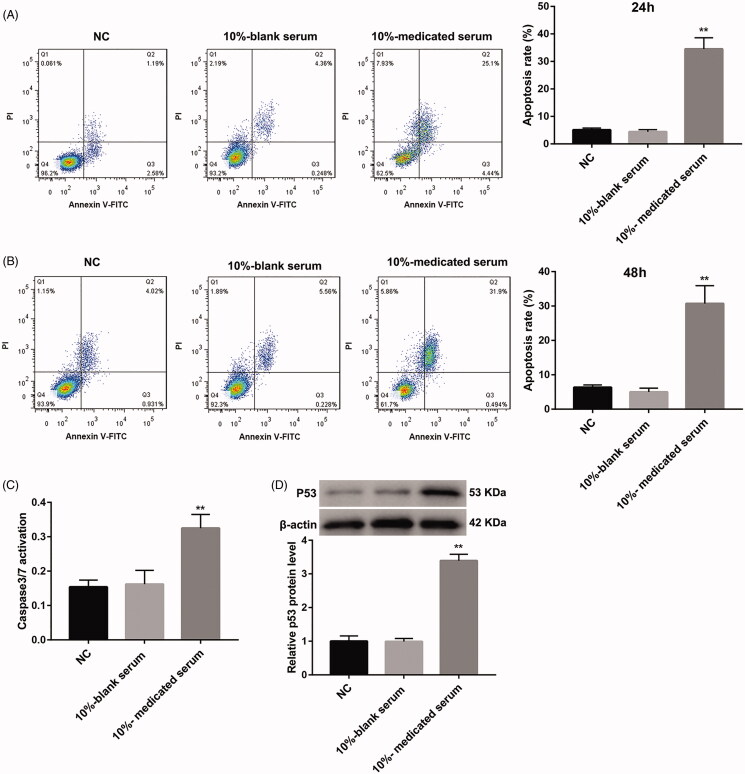
SQBD promotes apoptosis in CGM1 cells. CGM1 cells were treated with 10%-medicated serum or 10%-blank serum for 24 or 48 h. 10% FBS-treated CGM1 cells served as NC. (A and B) Flow cytometry was performed to detect apoptosis of the CGM1 cells after 24 or 48 h serum treatment. (C) The Caspase-Glo^®^ 3/7 assay was performed to detect the activity of caspase 3/7 in the CGM1 cells. (D) WB was performed to estimate the expression of p53 in the CGM1 cells. (**p < 0.01 *vs.* 10%-blank serum group).

We initially explored the effect of SQBD on cell viability of CGM1 cells. The data of CCK8 assay showed that 5%, 10% and 20%-medicated serum severely repressed the cell viability of CGM1 cells, especially 10%-medicated serum (Table 1). Thus, CGM1 cells were treated with 10%-medicated serum to detect the effect of the drug-medicated serum on apoptosis of CGM1 cells. Compared with the 10%-blank serum group, the apoptosis rate of CGM1 cells was remarkably increased after 24 or 48 h of 10%-medicated serum treatment (Figure 1(A,B)). Furthermore, we explored the activity of caspase 3/7 in the CGM1 cells, showing that the activity of caspase 3/7 was notably enhanced in the CGM1 cells after 10%-medicated serum treatment (Figure 1(C)). We also found that 10%-medicated serum treatment led to an up-regulation of p53 in the CGM1 cells (Figure 1(D)). Therefore, these data indicated that SQBD promoted apoptosis of CGM1 cells.

**Table 1.** Effect of the drug-contained serum on cell viability of CGM1 cells.

Groups	OD (24 h)	OD (48 h)	Inhibition ratio% (24 h)	Inhibition ratio% (48 h)
Control	0.35 ± 0.03	0.43 ± 0.01	–	–
5% - blank serum	0.44 ± 0.05	0.52 ± 0.02	–	–
5% - medicated serum	0.41 ± 0.04	0.50 ± 0.02	25.47 ± 6.98	21.62 ± 7.30
10% - blank serum	0.57 ± 0.04	0.82 ± 0.03		
10% - medicated serum	0.39 ± 0.05	0.55 ± 0.03	83 ± 26.07	69.88 ± 4.69
20% - blank serum	0.61 ± 0.04	0.85 ± 0.05	–	–
20% - medicated serum	0.48 ± 0.05	0.62 ± 0.04	51 ± 7.25	56.03 ± 2.56

### SQBD alters EBV latency protein expression in CGM1 cells

**Figure 2.**
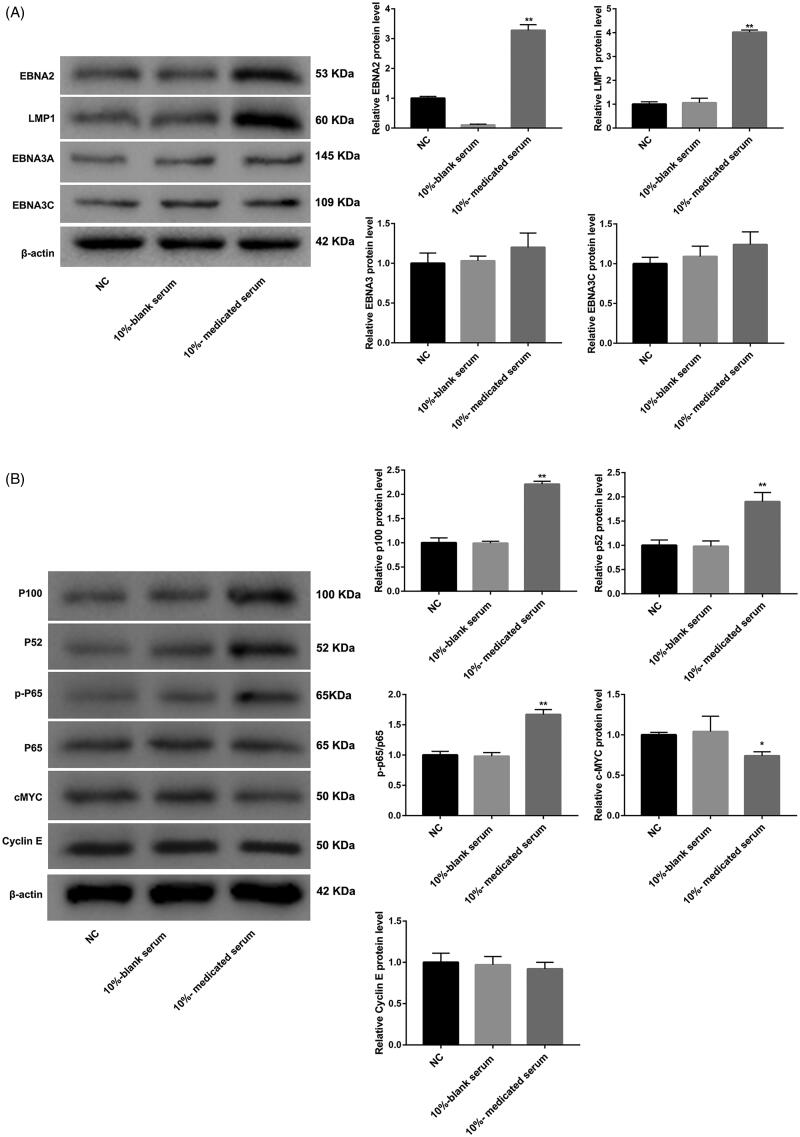
SQBD alters EBV latency protein expression in CGM1 cells. CGM1 cells were treated with 10%-medicated serum or 10%-blank serum. 10% FBS-treated CGM1 cells served as NC. (A) WB was performed to detect the expression of EBV latency proteins (EBNA2, LMP1, EBNA3A and EBNA3C) in the CGM1 cells. (B) WB was performed to assess the expression of p65, p-p65, p52, p100, c-MYC and cyclin E in the CGM1 cells. (*p < 0.05, **p < 0.01 *vs.* 10%-blank serum group).

In order to further probe into the role of SQBD in CGM1 cells, the expression of EBV latency proteins related to lymphoblastoid cell survival and proliferation were examined by WB. Compared with the 10%-blank serum group, the expression of EBV latency proteins, EBNA2 and LMP1, were highly expressed in the 10%-medicated serum group. However, the 10%-medicated serum treatment had no effect on the expression of EBV latency proteins, EBNA3A and EBNA3C, in the CGM1 cells (Figure 2(A)). LMP1 overexpression has an inhibiting effect on B cell proliferation (Kaykas and Sugden 2000). Taken together, these data suggested that LMP1 up-regulation may contribute to the anti-proliferative effect of SQBD in CGM1 cells. In addition, WB was performed to detect the activity of canonical or non-canonical NF-κB cell survival pathway in CGM1 cells after 10%-medicated serum treatment. NF-κB signalling induced by LMP1 is closely associated with EBV transformed lymphoblastoid cell survival (Ersing et al. 2013). The results showed that there was no different difference in the expression of p65 between 10%-blank and 10%-medicated serum groups. However, the expression of p-p65 (canonical NF-κB signalling marker) was notably increased in the CGM1 cells after 10%-medicated serum treatment. The expression of p52 and p100 (non-canonical NF-κB signalling markers) in the 10%-medicated serum group was higher than that in the 10%-blank group (Figure 2(B)). Thus, these data showed that the activity of NF-κB pathway was increased in CGM1 cells after 10%-medicated serum treatment. EBNA2-induced c-MYC expression is associated with EBV transformed lymphoblastoid cells proliferation (Zhao et al. 2011). Moreover, the expression of c-MYC was severely down-regulated in CGM1 cells after 10%-medicated serum treatment. However, there was no obvious influence on the expression of cyclin E, which was the target of c-MYC (Figure 2(B)). Taken together, these data showed that SQBD altered EBV latency protein expression in CGM1 cells.

### SQBD inhibits lytic EBV replication in CGM1 cells

**Figure 3.**
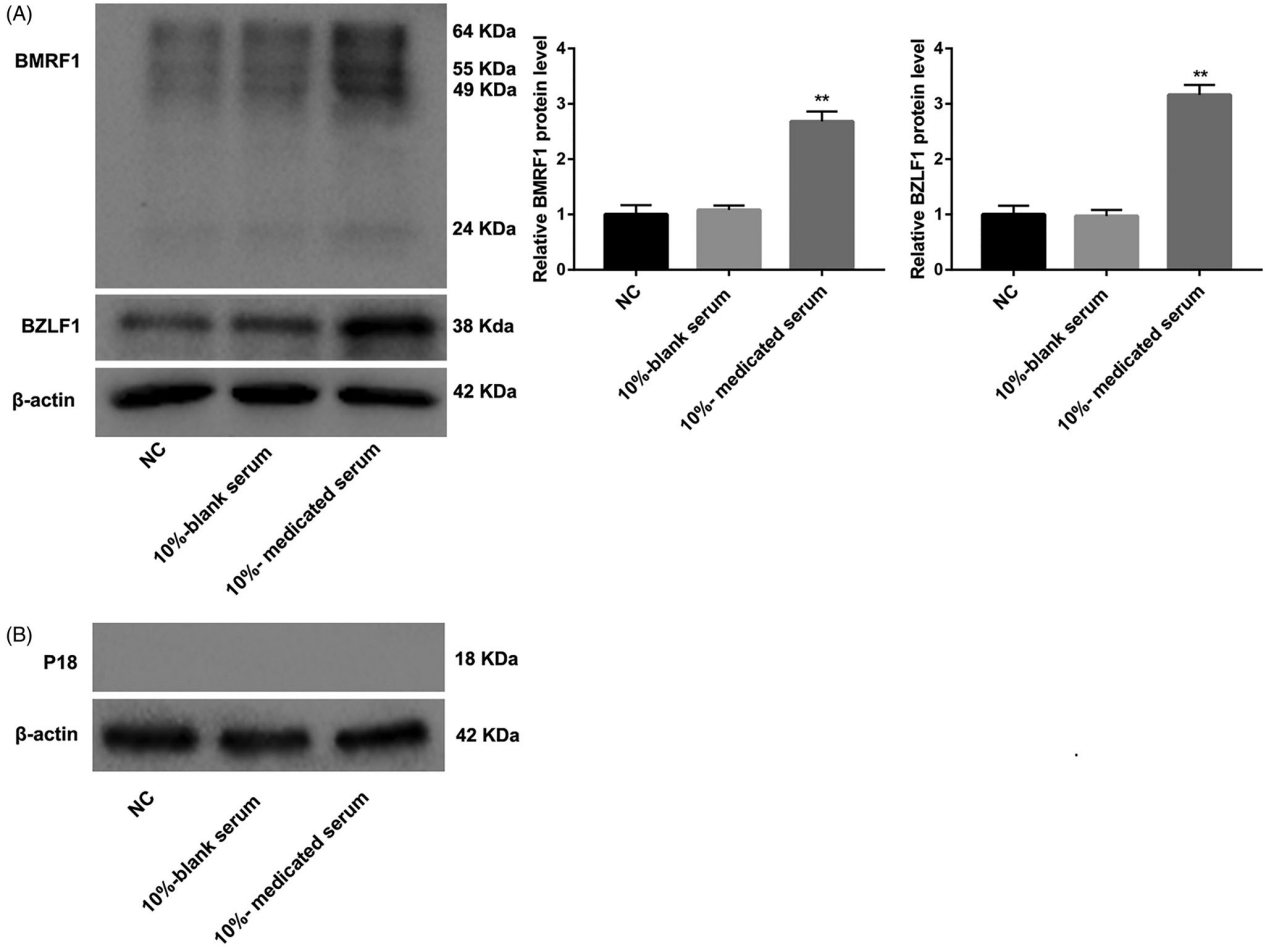
SQBD inhibits lytic EBV replication in CGM1 cells. CGM1 cells were treated with 10%-medicated serum or 10%-blank serum. 10% FBS-treated CGM1 cells served as NC. (A) WB was performed to explore the expression of immediate early lytic protein BZLF1 and the early lytic protein BMRF1 in the CGM1 cells. (B) WB was performed to assess the expression of p18 in the CGM1 cells. (**p < 0.01 *vs.* 10%-blank serum group).

The effect of SQBD on lytic EBV replication in CGM1 cells was detected by WB assay. Compared with the 10%-blank serum group, the expression of BZLF1 (immediate-early lytic protein) and BMRF1 (the early lytic protein) were significantly up-regulated in the 10%-medicated serum group (Figure 3(A)). However, there was no obvious difference in expression of p18 (late viral capsid antigen) between the 10%-blank serum group and the 10%-medicated serum group (Figure 3(B)). Therefore, these data suggested that lytic EBV replication in CGM1 cells was inhibited by SQBD treatment.

## Discussion

Previous studies have confirmed that leflunomide/teriflunomide represses lytic EBV infection through suppressing lytic viral reactivation and blocking lytic viral DNA replication (Bilger et al. 2017). In this work, we aimed to investigate the mechanism of action of Traditional Chinese medicine (SQBD) in preventing the EBV-induced lymphoproliferative disease. We found that different concentrations of SQBD-containing serum had inhibitory effects on cell viability of CGM1 cells, especially 10%-medicated serum. Thus, 10%-medicated serum was the most effective treatment concentration, and it was used to treat cells in subsequent experiments.

One study has revealed that some components of Qingwen Baidu decoction play an important role in the treatment of acute lung injury by reducing the total cells and infiltration of activated polymorphonuclear leukocytes, such as ethyl gallate, pentagalloylglucose, galloyl paeoniflorin, mudanpioside C and harpagoside (Zhang et al. 2017). Ethyl gallate and pentagalloylglucose of Qingwen Baidu decoction have a protective role in lipopolysaccharide-induced acute lung injury (Zhang, Li, et al. 2018; Zhang, Nie, et al. 2018). In our study, we initially explored that biological role of SQBD in EBV-induced B lymphoproliferative disease. We found that 10%-medicated serum treatment significantly promoted apoptosis of CGM1 cells. The activity levels of caspase 3/7 and p53 expression also enhanced in the CGM1 cells after treated with 10%-medicated serum. Tumour suppressor protein p53 plays a vital role in promoting both apoptosis and cell cycle arrest (Wawryk-Gawda et al. 2014). The activation of caspase 3/7 sets off explosive feedback amplification of upstream apoptotic events, which is a key feature of apoptotic signalling essential for efficient apoptosis (McComb et al. 2019). Thus, these data suggested that SQBD had a promoting effect on apoptosis of CGM1 cells.

In our study, the protein expression of LMP1 was significantly increased in CGM1 cells after treated with 10%-medicated serum. Previous study has reported that low level of EBV LMP, LMP1 promotes cell proliferation by activating NF-κB signalling pathway (Dirmeier et al. 2005). However, higher level of LMP1 expression has inhibiting effect on B cell proliferation (Kaykas and Sugden 2000). Therefore, SQBD may have an inhibiting effect on B cell proliferation by regulating LMP1 expression. Moreover, the protein expression of p-p65 (canonical NF-κB signalling marker), p52 and p100 (non-canonical NF-κB signalling markers) was significantly increased by 10%-medicated serum. 10%-medicated serum caused an up-regulation of EBNA2, and had no influence on the expression of EBV latency proteins EBNA3A and EBNA3C in CGM1 cells. Since EBV transformed lymphoblasts require LMP1-induced NF-κB for their survival (Ersing et al. 2013). EBNA2-induced c-MYC and cyclin E expression play an important role in lymphoblastoid cell line proliferation (Jansen-Durr et al. 1993; Zhao et al. 2011). Thus, these data showed that SQBD inhibited B cell proliferation by regulating LMP1-medicated canonical or non-canonical NF-κB signalling pathway, and it has no effect on c-MYC-mediated transcription in EBV-transformed B cells. In addition, we also found that SQBD caused an up-regulation of BZLF1 and BMRF1. However, SQBD had no effect on the expression of late viral capsid antigen p18. Therefore, these data suggested that lytic EBV replication in CGM1 cells was inhibited by SQBD treatment.

## Conclusions

SQBD promotes apoptosis of EBV-transformed B cells by regulating EBV latency protein expression. Therefore, SQBD inhibits EBV-induced B lymphoproliferative disease and lytic viral replication. Overall, our results suggest that SQBD could be a new treatment for EBV-induced B lymphoproliferative disease.
